# A comparative analysis of ESM-1 and vascular endothelial cell marker (CD34/CD105) expression on pituitary adenoma invasion

**DOI:** 10.1007/s11102-015-0698-6

**Published:** 2016-01-25

**Authors:** Yanming Miao, Miao Zong, Tao Jiang, Xuesen Yuan, Shusen Guan, Yisong Wang, Dabiao Zhou

**Affiliations:** Department of Neurosurgery, Beijing Tiantan Hospital, Capital Medical University, Beijing, China; Department of Neurosurgery, Beijing Electric Power Hospital, Capital Medical University, Beijing, China; Beijing Neurosurgical Institute, Capital Medical University, Beijing, China; Department of Microbiology, School of Basic Medical Sciences, Capital Medical University, Beijing, China

**Keywords:** Endocan, Pituitary adenoma, Tumor invasion, Immunohistochemistry

## Abstract

**Objective:**

Pituitary adenomas are benign neoplasms that display invasive behavior—a characteristic traditionally associated with malignancy—through an ill-defined mechanism. The role of angiogenesis-related molecules in this pathological condition remains perplexing. Our purpose is to assess the impact of endocan (endothelial cell specific molecule-1, ESM-1), CD34 and CD105 on pituitary adenoma invasion.

**Methods:**

In this study, immunohistochemical analyses for endocan, CD34 and CD105 were performed on paraffin-embedded samples of 66 pituitary adenomas, five normal pituitaries, and five primary hepatic carcinomas. Knosp tumor grades based on magnetic resonance imaging coronal scanning were used to assess the invasiveness of each sample. The associations between endocan expression, CD34/CD105-positive microvessel densities (MVDs), and Knosp tumor invasion grades were evaluated.

**Results:**

These results showed that endocan protein expression in tumor cells (TCs) was higher than that in endothelial cells (ECs) and strongly correlated with Knosp grades (*P* < 0.001, Spearman’s r = 0.616). Moreover, while endocan-positive TCs localized around the blood vessels in adenomas with higher Knosp grades, no significant association was found between CD34/CD105-MVDs and Knosp grades (CD34: *P* = 0.256, r = 0.142; CD105: *P* = 0.183, r = 0.166). Normal pituitary seemed to exhibit lower endocan expression and contained more CD34/CD105-MVDs than pituitary adenomas.

**Conclusion:**

Endocan expresses in both TCs and ECs of pituitary adenoma. Endocan overexpression in TCs more accurately reflects invasiveness compared to that of CD34/CD105-MVDs and that angiogenesis may not be the primary driver of endocan-medicated pituitary adenoma invasion.

## Introduction

Pituitary adenomas arise from intracranial adenohypophyseal cells and have a prevalence of 2.87–3.90 per 100,000 individuals in the general population [7, 23]. While histologically benign, approximately 35 % of pituitary adenomas exhibit aggressive or malignant growth patterns that can result in recurrence after initial surgical treatment [20]. Defined as invasive pituitary adenomas (IPA), they are inclined to invade cavernous sinus, the dura mater, bone, and even the central nervous system [2, 15]. Clinically, Knosp grades are based on the extent of invasion into the carotid artery and are a widely used measure of tumor aggression [10].

Angiogenesis plays an important role in a variety of tumor initiation and progression [6, 14, 17]. But its role in pituitary adenomas remains extremely controversial [2, 5, 14, 16, 18, 22, 24]. In previous investigations, several angiogenic factors or biomarkers—including CD34, endocan, and vascular endothelial growth factor (VEGF)—were found to be widely expressed in pituitary adenomas and associated with an aggressive phenotype [16, 18, 19]. In contrast, an increasing number of studies demonstrate that pituitary adenomas have lower angiogenesis and microvessel densities (MVDs) than normal pituitary tissues. Therefore, invasive pituitary adenomas may develop through a non-angiogenic process, which might explain why the majority of pituitary adenomas are less angiogenic than other tumor types [2, 5, 14, 22, 24].

Recently, expression of an intercellular signaling protein present in vascular endothelial cells, termed endocan (endothelial cell-specific molecule-1, ESM-1), was identified to be pivotal for tumor progression and invasion [1, 3, 16, 21, 27]. Endocan is exclusively expressed by CD34-positive vascular endothelial cells in pituitary adenomas, where it strongly correlates with an invasive phenotype [3, 16]. Although endocan overexpression by non-tumorigenic endothelial cells promotes tumor progression, accumulating evidence demonstrates that endocan may be of non-endothelial origin [3, 21]. Moreover, endocan expression was detected in several glioblastoma or renal carcinoma cell lines and primary human adipocytes [12, 17, 26]. but was rarely found in endothelial cells of normal pituitary tissues with the exception of a few isolated endocrine cells [3].

In the present study, we examined the expression of endocan, the pan-endothelial marker CD34, and the activated endothelial marker CD105 in pituitary adenomas and normal pituitary glands using semi-quantitative immunohistochemical staining. The purpose of this study was to clarify the expression pattern of endocan in pituitary adenomas and explore the association between endocan expression, CD34/CD105-MVD, and radiological Knosp grades to further assess the physiological role of endocan in pituitary tumor invasion.

## Methods

### Patients

Tumor samples were collected from a total of 127 patients with pituitary adenomas, who underwent transsphenoidal or craniotomy surgery (by Dabiao Zhou and Shusen Guan) at Beijing Tiantan Hospital between 2012 and 2014. All authors had obtained access to identifying information during data collection. Patients younger than 18 years old, with recurrent tumors, microadenomas (diameter <1 cm), or those who received medicine treatment or radiotherapy prior to surgical intervention were excluded from the study, leaving a total enrollment of 66 patients. The patient population consisted of 37 males and 29 females with an average age of 43.9 years old (range 18–68 years). Pre-operative examination consisted of endocrinology blood work, MRI with enhancement, and CT scans with bone window.

Five normal pituitary glands from deeded bodies without pituitary and endocrine disease and five primary hepatic carcinoma tissue samples were obtained from the Chinese Academy of Medical Sciences (CAMS) and Beijing Youan Hospital, respectively.

### Immunohistochemical staining

All tissues were fixed in 10 % formaldehyde for 24 h, embedded in paraffin and cut into 4-μm-thick sections. After incubation in a thermo tank for 30 min, the sections were deparaffinized and subjected to immunohistochemistry. The slides were then rinsed in distilled water for 2 min and treated with 3 % hydrogen peroxide for 12 min to suppress endogenous peroxidases. Next, the slides were rinsed with distilled water for 5 min, washed in PBS (phosphate buffer saline) three times for 5 min each, soaked in a citrate buffer (0.01 M, pH 6.0), heated in a microwave oven, naturally cooled to room temperature, washed once in distilled water and washed in PBS twice for 5 min each for antigen revival. After a 30 min incubation in PBS containing 2 % BSA, the slides were stained with primary antibodies—including mouse monoclonal endocan (ab56914, 1:1000; Abcam, Cambridge, MA, USA), mouse monoclonal CD34 (sc-19621, 1:250; Santa Cruz, Dallas, TX, USA), and goat monoclonal CD105 (sc-23838, 1:250; Santa Cruz)—and incubated overnight at 4 °C. The next day, the slides were rewarmed for 30 min at room temperature, washed twice in PBS for 5 min each, incubated in polymer helper (pv9002; Beijing Zhongshan Golden bridge Biotechnology, Beijing, China) for 20 min at room temperature, washed again in PBS twice for 5 min each, and then allowed to react with the corresponding secondary antibody solution for 20 min at room temperature. After two washes in PBS for 5 min each time, the slide were visualized using 0.05 % diaminobenzedinetetrahydrochloride (DAB, ZLI-9018; Beijing Zhongshan Golden Bridge Biotechnology, Beijing, China), washed in tap water, rinsed in distilled water for 5 min, incubated with hematoxylin, washed in tap water, rinsed in 75 % alcohol and 0.5 % hydrochloric acid, dehydrated, cleared, and then mounted with neutral gum.

### Evaluation of the cytoplasm staining reaction and determination of the MVD

For evaluation of endocan, the immunoreactive score (IRS) was obtained by multiplying the staining intensity (SI: 0 = negative; 1 = weak; 2 = intermediate; 3 = strong) and percentage of positive tumor/endothelial cells (PPC: 0 = 0 %; 1 = 1–10 %; 2 = 11–50 %, 3 = 51–80 %; 4 = >80 %) as established previously 24. IRS = SI × PPC. Five high power fields (HPFs) (400×) were randomly selected from each slide for IRS calculation. To determine the MVD, tissue sections stained with CD105 and CD34 were examined using an Olympus BX61 light microscope. According to the methods exposed by Weidner previously [25]. The sections were firstly examined at 100× magnification for the location rich of blood vessels. Then, pictures of five different fields were taken at 200× magnification. Finally, the number of blood vessels in each picture was counted independently by two researchers. Every single cell and cell cluster stained were assessed as a blood vessel, regardless of whether the structure of vascular lumen was observed. Every 40 microns of one large lumen was recorded as one blood vessel. The average value of five fields was recorded as the MVD score [25].

### Radiological assessment for cavernous sinus invasion

The intercarotid lines described by Knosp et al. on magnetic resonance imaging (MRI) coronal scanning were used to assess cavernous sinus invasion for the 66 tumors. Five grades are used to represent the extent of tumor invasion from Grade 0 to 4. According to the Knosp MRI grading system, Grade 3, Grade 4, and >80 % of Grade 2 pituitary adenomas were considered to be invasive [10].

### Statistical analysis

Statistical analysis was performed in SPSS 16.0, JMP 10.0, and Graphpad 6.04. Rank sum tests were used to estimate the difference of IRS between tumor cells and endothelial cells. Spearman’s correlation was used to assess the relationships between endocan IRS, CD34/CD105-MVD, and Knosp tumor grades. Pearson correlation was used to assess the relationships between endocan IRS and CD34/CD105-MVD. Statistical significance was defined as *P* < 0.05.

### Ethical permission

The current study was approved by the Clinical Research Ethics Committee of CAMS (Project No: 009-2014). All patient records were anonymized prior to analysis and were approved for use by Clinical Research Ethics Committee of Beijing Tiantan Hospital (Project No: KY2014-021-02).

## Results

### Patient population and immunohistochemical data

Of the 66 pituitary adenomas in the present study, 31 (47.0 %) were functioning adenomas [15 prolactinomas, five growth hormone (GH), one adrenocorticotrophic hormone (ACTH) adenoma, ten mixed adenomas], 35 (53.0 %) were non-functioning ademomas (26 null cell adenomas and 9 gonadotrophic adenomas). Table 1 summarizes the distribution of Knosp grades across the patient population and the corresponding endocan expression IRS in tumor/endothelial cells, as well as CD34/CD105-positive MVD. Five pituitary tissues were immunostained for endocan and CD34/CD105 as controls.

**Table 1:** Knosp tumor grades, mean endocan IRS, and mean MVD score for CD34/CD105-positive capillaries in 66 pituitary adenomas

Knosp grade	No.	Mean IRS-TCs	Mean IRS-ECs	MVD	
				CD34 (Mean ± SD)	CD105 (Mean ± SD)
0	13	4.8	1.8	44.3 ± 30.1	40.4 ± 23.4
1	10	4.1	4.4	46.0 ± 22.2	44.7 ± 18.1
2	15	6.8	4.9	50.4 ± 30.1	51.6 ± 28.4
3	13	9.2	4.2	60.8 ± 30.6	55.3 ± 31.9
4	15	10.2	4.3	48.3 ± 17.9	47.6 ± 16.7
Total	66	7.2*	3.9	50.1 ± 26.6	48.2 ± 24.4
Pituitary	5	5.2	3.0	72.1 ± 9.7	136.8 ± 35.4

*TCs* tumor cells, *ECs* endothelial cells

* In 66 pituitary adenomas, tumor cells IRS was significant higher than vascular endothelial cell IRS (*P* < 0.001)

### Endocan immunoreactivity in tumor cells and vascular endothelial cells of pituitary adenomas

**Fig. 1:**
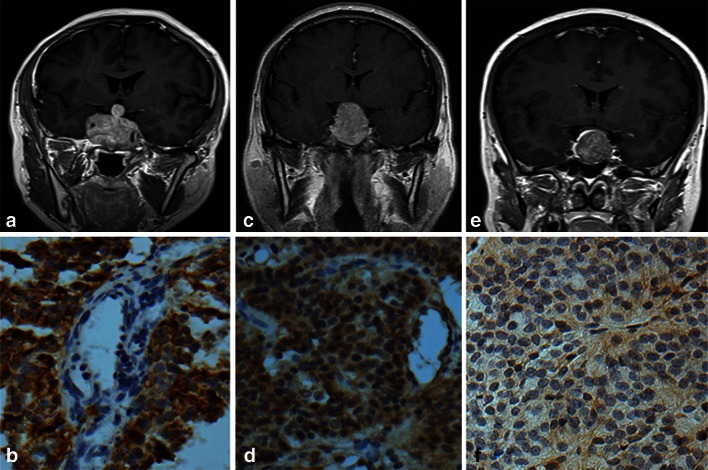
Coronal MR images with enhancement and endocan expression in the Knosp Grade 4 (**a**, **b**), 2 (**c**, **d**), and 0 (**e**, **f**) pituitary adenomas. In Grade 4 pituitary adenoma, ICA were entirely encased by tumor (**a**) and endocan was expressed in the cytoplasm of tumor cells and a few endothelial cells (**b**; IRS-TC = 12, IRS-EC = 1). Endocan expression was weaker in Grade 2 tumor which crossed the ICA center line but not the tangent line temporal side of blood vessel (**c**). Although endocan was expressed in tumor cells and endothelial cells, some cells were scattered in a negative expression (**d**; IRS-TC = 8, IRS-EC = 6). The Knosp Grade 0 adenoma did not cross the tangent line nasal side of blood vessel (**e**) and exhibited lower endocan reactivity (**f**; IRS-TC = 1, IRS-EC = 1). Magnification, ×400

**Fig. 2:**
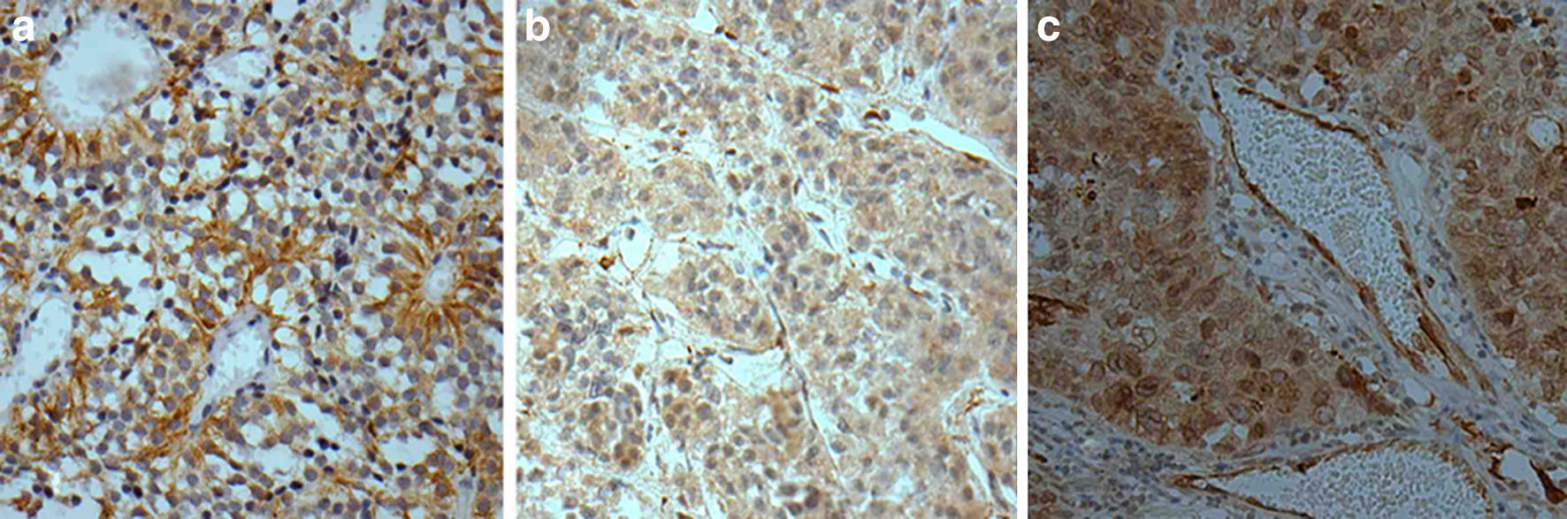
Endocan expression in pituitary adenoma, normal pituitary and primary hepatic carcinoma. Endocan was overexpressed in tumor cells and a few vascular endothelial cells in Grade 4 pituitary adenomas. Tumor cells surrounded the blood vessel intensely, with endocan characteristically distributed in the cytoplasm and polarized towards the blood vessel lumen (**a**). Endocan was weakly expressed in endocrine cells and a few vascular endothelial cells in the normal pituitary gland (**b**). In primary hepatic carcinoma, endocan was over-expressed in vascular endothelial cells within the cancer nests and adjacent tumor cells (**c**). Magnification: A, ×400; B, ×100; C, ×400

Immunohistochemical staining of pituitary adenoma tissues revealed that endocan was mostly expressed specifically in tumor cells and a few vascular endothelial cells (Fig. 1a–e). Quantitatively, endocan expression in tumor cells had a significantly higher immunoreactivity score than that of vascular endothelial cells (*P* < 0.01, Table 1). In Knosp Grade 3 and 4 tumors, endocan was often detected in the cytoplasm of tumor cells surrounding the blood vessels with a high polarity toward the vessel lumen side (Fig. 2a). In the normal pituitary gland, endocan was weakly expressed in endocrine cells and a few vascular endothelial cells (Fig. 2b), whereas it was mainly expressed in the vascular endothelial cells in cancer nests and adjacent tumor cells in primary hepatic carcinoma samples (Fig. 2c).

### Correlation between endocan expression and Knosp tumor grades

**Fig. 3:**
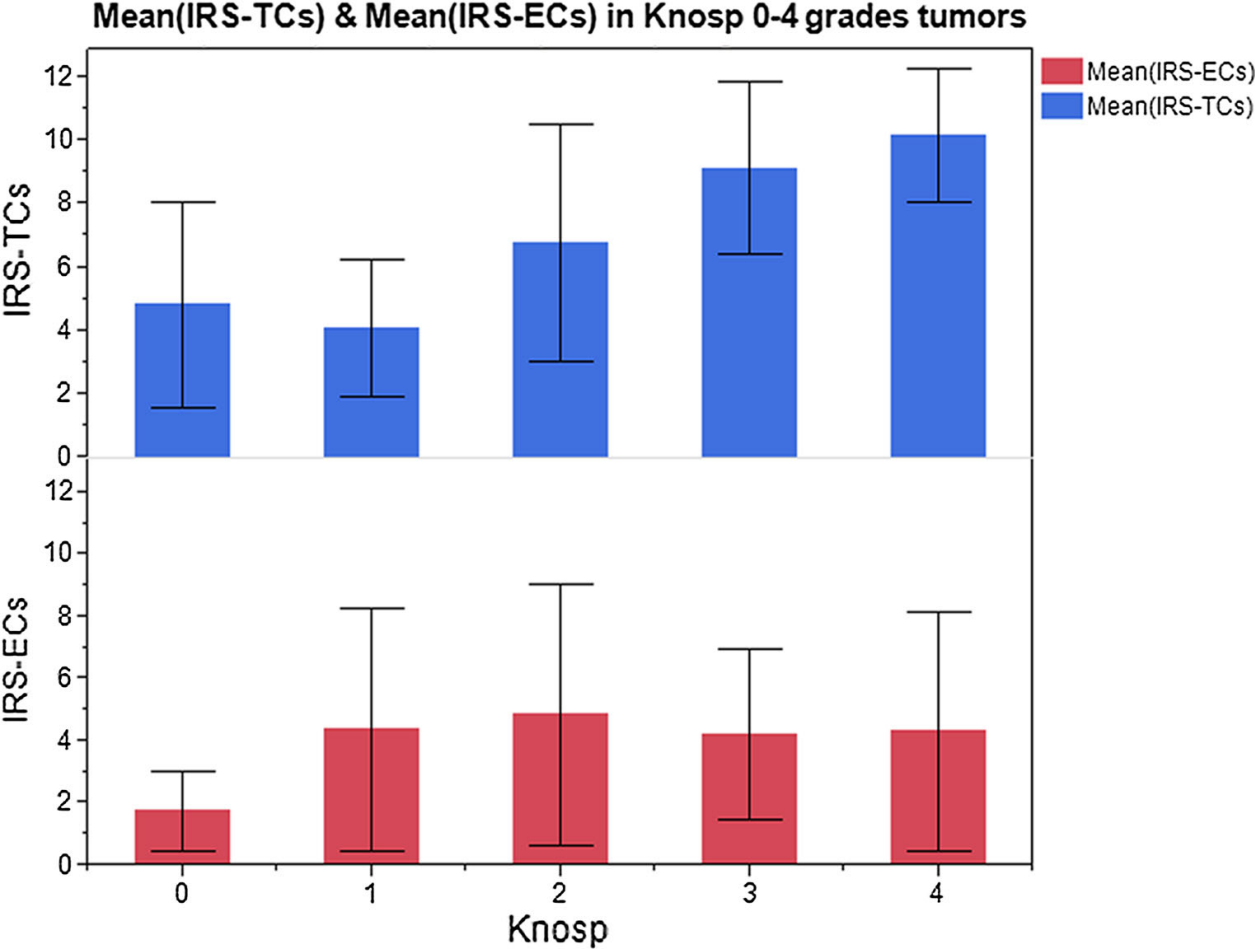
Distribution of endocan IRS in tumor cells (IRS-TCs) and endothelial cells (IRS-ECs) of five Knosp tumor grades

The distribution of the IRS (tumor and endothelial cells) in pituitary adenomas according to Knosp tumor grades are shown in Fig. 3. The mean IRS for endocan with respect to each Knosp grade was 4.8 (ranging from 1 to 12) for Grade 0, 4.1 (1–6) for Grade 1, 6.8 (1–12) for Grade 2, 9.2 (4–12) for Grade 3, and 10.2 (6–12) for Grade 4 (Table 1). Spearman’s rank correlation test demonstrated a significant and positive correlation of endocan expression in tumor cells with Knosp tumor grade (*P* < 0.001, Spearman’s r = 0.616). In comparison, endocan expression in endothelial cells was unrelated to Knosp tumor grades (*P* > 0.05, Spearman’s r = 0.208; Linear regression, r^2^ = 0.3679 (Knosp and TCs), r^2^ = 0.0433 (Knosp and ECs).

### CD34 and CD105 expression in pituitary adenomas and normal glands

**Fig. 4:**
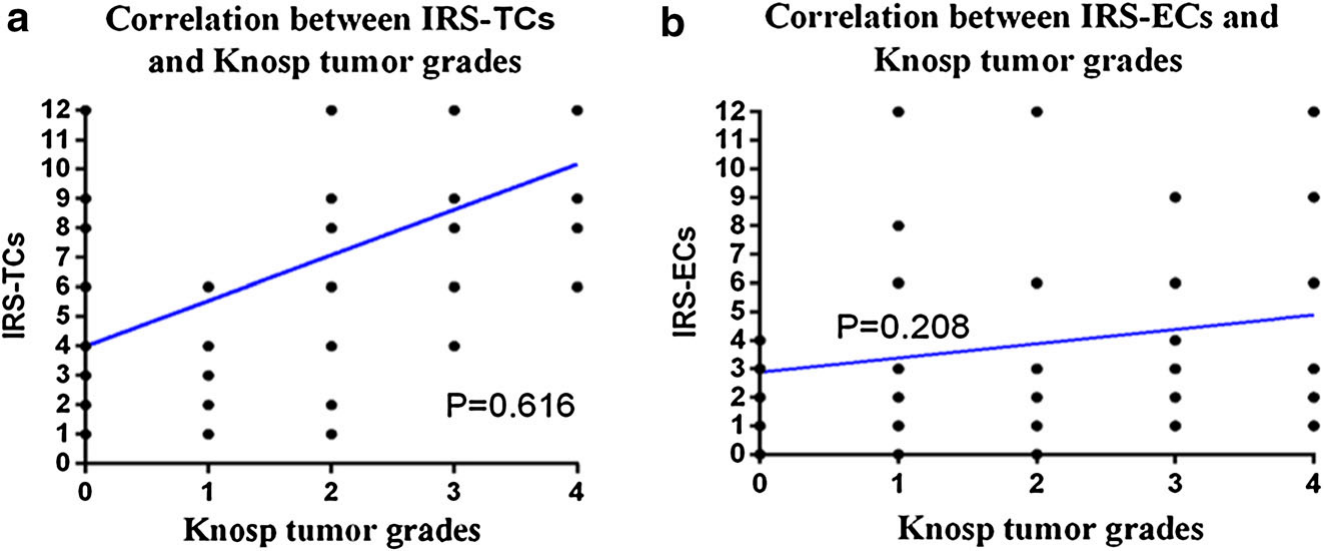
The tendency of 66 endocan immunoreactivity in tumor cells (IRS-TCs)/endothelial cells (IRS-ECs) according to Knosp tumor grades. Correlations between endocan immunoreactive score (IRS) in TCs/ECs and Knosp tumor grades (**a** *P* < 0.001, Spearman’s r = 0.616; **b** *P* > 0.05, Spearman’s r = 0.208)

**Fig. 5:**
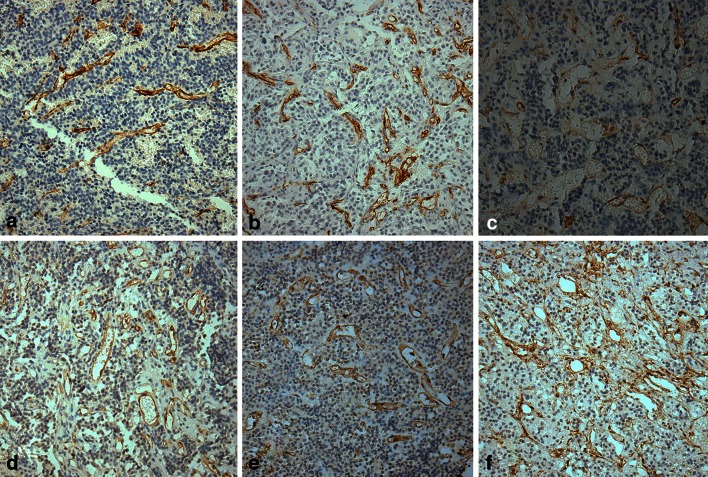
Tissues from representative IRS-TC = 12 (**a**, **d**) and IRS-TC = 1 (**b**, **e**) pituitary adenomas and from a normal pituitary gland(**c**, **f**) for CD34 (**a**–**c**) and CD105 (**d**–**f**) immunostaining. MVDs in panels **a** and **d** are not more noticeable than those in **b** and **e**, whereas those in the normal pituitary gland are the most pronounced in panels **c** and **f**. Magnification, ×200

CD34 and CD105 immunoreactivity was exclusively observed in vessel endothelial cells. Representative examples of vessels stained for CD34 and CD105 in pituitary adenomas and normal pituitary glands are shown in Figs. 4 and 5. Interestingly, while MVDs in the pituitary adenomas with high endocan expression were slightly higher than those observed in tumors with low expression, MVDs were the most distinct in the normal pituitary gland (Table 1).

### Correlation between CD34/CD105-MVDs and Knosp tumor grades

The mean positive MVD based on CD34/CD105 immunostaining under 200× magnifications for each Knosp grade was shown in Table 1. No significant association was observed between Knosp tumor grades and the presence of CD34- or CD105-positive MVDs (CD34: *P* = 0.256, Spearman’s r = 0.142; CD105: *P* = 0.183, Spearman’s r = 0.166) (Table 1).

### Correlation between tumor cell endocan expression and CD34/CD105-MVDs

Pearson’s rank correlation testing demonstrated a significant, albeit weak, correlation between CD34/CD105-MVDs and endocan expression in tumor cells (CD34: *P* = 0.034, Pearson’s r = 0.262; CD105: *P* < 0.010, Pearson’s r = 0.316).

## Discussion

Pituitary adenomas with clinically aggressive phenotypes carry a lower ratio of total resection and higher recurrence rate after surgical intervention. Much effort has focused on the identification of key molecules that regulate tumor growth and invasion. Recently, endocan (endothelial cell-specific molecule-1, ESM-1) secreted by endothelial cells was found to associate with tumor development and progression [1, 3, 16, 21, 27].

Endocan is a soluble, 50-kDa proteoglycan comprising a core protein that consists of 165 amino acids and a single dermatan sulfate chain. Endocan was originally cloned from a human endothelial cell cDNA library [11]. As a biomarker of neovascularization, increased endocan expression is often used as an indicator of tumor progression and metastasis in malignances such as glioblastoma, non-small cell lung cancer, renal cell carcinoma, hepatocellular carcinoma and others [8, 9, 12, 17]. In addition, endocan is also found to be strongly associated with tumor invasion in benign pituitary adenomas [3, 16]. Like other proteoglycans, the biological characteristics of endocan depend upon the binding activity through either the core protein or dermatan sulfate chain. In an early study in a xenotransplantation mouse model of colon cancer, endocan overexpression in HEK293 and HT29 cells found to enhance tumorigenesis, validating endocan as a protumorigenic factor [3].

Here, a total of 66 pituitary adenomas, five normal pituitary glands, and five primary hepatic carcinoma samples were examined for endocan expression by immunohistochemistry. We found that endocan expression—especially that observed in tumor cells—was strongly associated with Knosp tumor grades. While both endothelial and tumor cells expressed endocan in pituitary adenomas, there were many endocan-negative endothelial cells in both invasive and non-invasive tumors. Our results are similar to the findings in cultured cell lines in which endocan was found to be expressed in tumor cells, such as human glioblastomas and renal carcinoma, as well as human adipocytes [12, 17, 26]. These endocan-positive cells are of non-endothelial origin. In hepatocellular carcinoma tissues, Kang et al. [9] observed strong ESM-1 expression in the cytoplasm of tumor cells compared to normal tissues by immunofluorescent staining. Moreover, in a study of 159 gastric carcinomas, ESM-1 protein was detected in the tumor epithelium in more than half of samples, particularly in the tumor cell plasma membrane [13]. Using immunohistochemistry, we first revealed that endocan is mainly synthesized and secreted by tumor cells in pituitary adenomas. Additionally, endocan-positive tumor cells were found to adhere to the blood vessels intensely in tumors with higher Knosp grades. Collectively, we theorize that increased endocan expression in tumor epithelial cells, rather than the vascular endothelial cells, may be an essential step in tumor formation and growth, and is therefore associated with aggressive behavior.

Angiogenesis is considered to be critical for malignant tumor progression, but this notion has raised much controversy in the analysis of pituitary tumors [2, 14, 16, 18, 24]. A number of studies revealed that pituitary adenomas have lower MVDs than normal pituitary tissues, and pituitary adenomas commonly display less angiogenesis than other tumor types [2, 5, 14, 22, 24]. Consistent with this fact, we also found that the mean positive MVDs of CD34 and CD105 in the five normal pituitary glands were markedly higher than those in pituitary adenomas, regardless of the Knosp tumor grade. It was thus speculated by our groups that lower vascularization in pituitary adenomas may be basis for the lower occurrence of tumor malignant transformation or metastasis, although some adenomas are clinically invasive.

In the fluorescence analysis of 70 pituitary adenomas, Matano et al. indicated that expression was detected in all vascular endothelial cells, but in very few tumor parenchymal cells; whereas endocan localized with more than 90 % of CD34-positive endothelial cells in the samples examined [16]. Thus, they concluded that a significant relationship exists between endocan expression, Knosp tumor grades, and CD34-positive vessels of pituitary adenomas; however, we were unable to find any significant association between the presence of CD34- or CD105-MVDs and Knosp tumor grades. Rather, our results show that endocan expression in tumor cells more accurately reflects the invasiveness when compared to that of CD34/CD105-positive MVDs. We believe that this was not a sequence of different immunostaining methods or techniques. We support the statement in a review article that endocan is a protumorigenic molecule when overexpressed in tumor epithelial cells [4].

There are limitations in the present study. A given tumor’s Knosp grade may be dependent on the time of imaging (i.e., there is a wide variability of IRS scores from 0 to 12 for Knosp Grade 0 tumors). The pathophysiological significance of our finding should be confirmed clinically by follow up for tumor recurrence. Further study should address on insight into the mechanism regulated by endocan and other growth/vascular factors, cytokines, signal factors in tumor epithelium and endothelial cells has the potential to further the development of invasive pituitary tumor therapies.

## Conclusion

We found that endocan protein expression was mainly detected in the tumor cells of pituitary adenomas and was significantly correlated with Knosp grades when compared to that of CD34/CD105-positive MVDs. These results suggested that endocan-mediated tumor progression occurs through an uncertain mechanism, but is likely dependent on endocan binding to or interacting with multiple molecules, rather than directly promoting angiogenesis. Endocan overexpression strongly correlated with the invasive behavior of pituitary adenomas, which have less angiogenesis than normal pituitary tissues.
